# Intraepithelial Lymphocytes and LAIR1 Expression in Celiac Disease

**DOI:** 10.3390/biomedicines13102526

**Published:** 2025-10-16

**Authors:** Joaquim Carreras, Giovanna Roncador, Rifat Hamoudi, Jose Antoni Bombi, Yohei Masugi

**Affiliations:** 1 Department of Pathology, School of Medicine, Tokai University, 143 Shimokasuya, Isehara 259-1193, Japan; masugi@tokai.ac.jp; 2 Monoclonal Antibodies Core Unit, Spanish National Cancer Research Center (CNIO), Melchor Fernandez Almagro 3, 28029 Madrid, Spain; groncador@cnio.es; 3 Research Institute for Medical and Health Science, Department of Clinical Sciences, College of Medicine, University of Sharjah, Sharjah P.O. Box 27272, United Arab Emirates; rhamoudi@sharjah.ac.ae; 4 Division of Surgery and Interventional Science, University College London, London W1W 7TY, UK; 5 Biomedically Informed Artificial Intelligence Laboratory (BIMAI-Lab), University of Sharjah, Sharjah P.O. Box 27272, United Arab Emirates; 6 Center of Excellence for Precision Medicine, University of Sharjah, Sharjah P.O. Box 27272, United Arab Emirates; 7 ASPIRE Precision Medicine Research Institute Abu Dhabi, University of Sharjah, Sharjah P.O. Box 27272, United Arab Emirates; 8 Department of Pathology, Faculty of Medicine, Reial Academia de Medicina de Catalunya, University of Barcelona, Campus Clinic, Casanova, 143, 08036 Barcelona, Spain; bombi@ub.edu

**Keywords:** small intestine, celiac disease, intraepithelial lymphocytes, immune-phenotype, immuno-oncology, LAIR1, BTLA, artificial intelligence, deep learning, convolutional neural network

## Abstract

**Background**: Celiac disease (CD) is a gluten-sensitive immune-related enteropathy of the small intestine characterized by villus atrophy, crypt hyperplasia, and increased intraepithelial lymphocytes (IELs). **Objectives**: To characterize the phenotype of IELs and immune cells of the lamina propria of small intestine control using immuno-oncology and immune-phenotype markers and test the most relevant marker, an immune checkpoint co-inhibitory receptor, leukocyte-associated immunoglobulin-like receptor 1 (LAIR1), in CD. **Methods**: Immunohistochemical analysis of CD3 (CD3E), CD4, CD8, CD103 (ITGAE), Granzyme B (GZMB), TCR beta (β), TCR delta (δ), CD56 (NCAM), CD16 (FCGR3A), LAIR1 (CD305), PD-L1 (CD274), PD1 (CD279), BTLA (CD272), TOX2, HVEM (TNFRSF14), CD163, HLA-DP-DQ-DR, IL4I1, and FOXP3 was performed using histological analysis. Gene expression analysis was performed using an independent dataset to expand and confirm the findings. **Results**: IELs exhibited a cytotoxic T-cell phenotype and were CD3+, CD8+, CD103+, TCR beta+, and LAIR1+. The lamina propria (LP) was abundant in CD163+, HLA-DP-DQ-DR+, BTLA+, PD-L1+, CD103+, CD56+, and LAIR1+ cells corresponding to macrophages and T- and B-lymphocytes. In CD, IELs and part of the inflammatory cells of the lamina propria cells were LAIR1+. CD was characterized by higher quantity of LAIR1+ IELs and LP immune cells than the small intestine control (*p* = 0.004). Higher intestinal lesions evaluated by Marsh scoring were correlated with higher LAIR1 (*p* < 0.001). Gene expression analysis confirmed the overexpression of the *LAIR1* pathway in CD and highlighted *BTLA*. At the protein level, BTLA overexpression was confirmed in CD. Finally, as a proof-of-concept AI analysis, a convolutional neural network classified LAIR1-stained image patches between the three diagnoses of small intestine control, CD, and reactive tonsils with high accuracy (99.6%). **Conclusions**: IELs exhibit a cytotoxic T-cell phenotype and were found to be CD3+, CD8+, CD103+, TCR beta+, and LAIR1+ in the small intestine control. Increased numbers of LAIR1+ IELs and lamina propria immune cells characterize CD.

## 1. Introduction

### 1.1. Histology of the Small Intestine

The normal intestinal mucosa has a defined architecture, including the villi, crypts, lamina propria, and muscularis mucosae.

The villi exhibit a digitiform shape with a 3:1 ratio between the height of the villi and the depth of the glandular crypts. The glandular crypts comprise several cell subtypes, including epithelial, Paneth, goblet, and endocrine cells. Each of these cells has different functions.

Intestinal epithelial cells (IECs) line the surface of the intestine and are responsible for aliment digestion, nutrient absorption, and infection protection by creating a physical barrier and modulating the immune response [1]. Intestinal epithelial cells are sensitive to the nutrients in the diet [1]. Paneth cells secrete alfa defensins, which are broad-spectrum microbicides that control gut microbiota and intestinal homeostasis. In hematoxylin and eosin (H&E) staining, Paneth cells display bright red cytoplasmic granules [2]. Goblet cells produce mucus and are intimately involved in controlling the mucosal immune system [3]. Goblet cells sample luminal antigens to initiate the adaptive immune response. There are several subtypes of goblet cells with different localization and gene expression [3]. There are several types of endocrine cells in the small intestine [4]: EC cells produce serotonin (5-HT) [5,6,7]; L cells produce GLP-1, GLP-2, and peptide YY [8,9,10]; K cells produce GIP and 5-HT [11,12]; I cells produce cholecystokinin and 5-HT [13,14]; D cells produce somatostatin [15,16]; G cells produce gastrin [17,18,19]; N cells produce neurotensin [20,21]; M cells produce motilin [22,23]; and S cells produce secretin [24,25] (Table 1). The main functions of endocrine cells are gut motility, appetite control, insulin release, cell proliferation control, gastric acid motility, pancreatic enzyme secretion, and intestinal absorption [4].

The lamina propria is a thin connective tissue layer located below the epithelial basement membrane. The lamina propria is rich in fibroblasts, myofibroblasts, vascular and lymphatic vessels, elastic fibers, smooth muscle fascicles, and immune cells, including lymphocytes, plasma cells, macrophages, eosinophils, and mast cells [26].

The muscularis mucosa is composed of a very thin layer of smooth muscular cells with motor activity, which are linked to mucosal absorption and secretion functions [27].

The submucosa contains blood and lymphatic vessels and nerves of the parasympathetic system, including the submucous plexus, also known as Meissner’s plexus [28]. The submucosal extracellular matrix is minimally immunogenic [28]. The muscularis propria [29] comprises an inner circular and outer longitudinal layer, and Auerbach’s (myenteric) plexus.

### 1.2. Instraepithelial Lymphocytes

IELs are found in the epithelium of the skin, genitourinary tract, respiratory tract, and intestinal tract [30]. IELs are a first line of defense against pathogens that have attacked the epithelial surface. The typical phenotype is of cytotoxic T-lymphocytes, being CD3- and CD8-positive [31]. The T-cell receptor (TCR) can be alphabeta (αβ)-positive or gammadelta (γδ)-positive. Some IELs present with self-reactive TCR, suggesting an extrathymic origin [30,32,33,34,35,36].

IELs are specialized immune cells that colonize the intestinal mucosa. Although B and innate cell populations may also transit inside this compartment, T-lymphocytes comprise the majority of intestinal IELs. IELs represent one of the largest lymphocyte populations in the intestine and contribute to epithelial homeostasis and barrier integrity, including tolerance, resistance, and tissue protection [37]. There are several subsets of IELs. However, all strains share common characteristics, including restricted TCR diversity, an epithelium-adapted profile, innate-like properties, and cytotoxic potential [37]. Human IELs can recognize modified self-antigens using both natural killer (NK) receptors and foreign antigens using the TCR [31]. The main characteristics of IELs are as follows:

(1) IELs permanently reside in the epithelial tissue and do not recirculate because of the expression of CD103 [38,39] that binds to E-cadherin [39,40,41]. CD103 is also known as ITGAE (Integrin, Alpha E, and Human Mucosal Lymphocyte Antigen 1). E-cadherin is also known as Cadherin-1 (CDH1), and CD324.
(2) The mucosal epithelial environment is highly immunogenic, with constant activation and tolerance that prevents tissue damage. Therefore, IELs express several T-cell co-inhibitory molecules and NK inhibitory receptors [42,43] and downregulate TCR co-stimulatory molecules.
(3) The TCR diversity of IELs is limited compared to peripheral T-lymphocytes [44,45] and specific to conserved microbial or dietary antigens [46].
(4) IELs have innate-like properties enabling rapid TCR-independent responses to stress signals [42,47].
(5) IELs have cytotoxic activity [47,48,49,50], and an alteration may be associated with several gastrointestinal diseases such as celiac disease and inflammatory bowel disease (IBD) [50,51,52,53,54].
(6) IELs are stratified into natural IELs (nIELs) and peripherally induced IELs (pIELs) [55,56,57,58]. nIELs are generated in the thymus and migrate to the intestine. In contrast, pIELs are derived from CD4-positive or CD8-positive T cells at inductive sites, such as gut-associated lymph nodes, in response to dietary and microbial antigens [31,37,55,56,57,58,59,60,61].
(7) IELs can be further subclassified according to their TCR subtype: (I) TCRγδ + nIELs (tissue surveillance and repair), (II) TCRαβ + CD8αα + nIELs (regulation), (III) TCRαβ + CD8αβ + pIELs (effector memory, cytotoxicity), (IV) TCRαβ + CD4 + pIELs (regulation, cytotoxicity) [31,37]. Subtypes I and II may recognize self-antigens using their TCR, are present at birth, and are microbiota-independent. Subtypes III and IV may recognize microbial, viral, and dietary antigens using TCRs, are absent at birth, increase with age, and are microbiota- and diet-dependent [31,37]. CD4 + FOXP3 + regulatory T-lymphocytes (Tregs) can undergo CD4 + CD8αα + IEL differentiation in the intestinal epithelium [62,63].
(8) CD8αα+ is an indication of intestinal IELs. Conventional CD8 + T cells express the CD8αβ heterodimer that is a TCR coreceptor and enhance TCR-MHC-I interactions during antigen presentation. Most IELs express CD8αα homodimer that decreases TCR sensitivity and prevents IEL hyperactivation via the mechanism of CD8αα homodimer interaction with thymus leukemia (TL) antigen [64], which is expressed by intestinal epithelial cells. Therefore, TL expression plays a critical role in maintaining IEL effector functions. In a genetic model of inflammatory bowel disease, TL deficiency was associated with colitis [65].
(9) IELs contribute to chronic intestinal inflammatory disease pathogenesis. Inflammatory bowel disease (IBD) includes Crohn disease and ulcerative colitis. Dysregulated intestinal immune response to microbiota is a cause of IBD [66,67]. IELs could play a regulatory role in IBD [65,66,67,68,69,70,71,72]. Preserved villous architecture and increased IELs characterize microscopic colitis [73,74,75,76]. Celiac disease is an autoimmune disease triggered by dietary gliadin and is characterized by villous atrophy, crypt hyperplasia, and chronic inflammation of the lamina propria [77,78,79,80]. In celiac disease, there are increased CD8αβ+ pIELs and TCRγδ+ nIELs [31]. IELs can undergo neoplastic transformation into enteropathy-associated T-cell lymphoma, a rare complication in patients with celiac disease who are unresponsive to gluten-free diet and treatment [81,82,83,84] (Table 2).

### 1.3. Celiac Disease

Celiac disease is a common immune-related disease with a prevalence of approximately 1% in most populations [85]. The incidence of celiac disease has increased in recent years; the reason for this is unknown, but it may be related to environmental factors associated with the loss of tolerance to dietary gluten [85].

**Figure 1:**
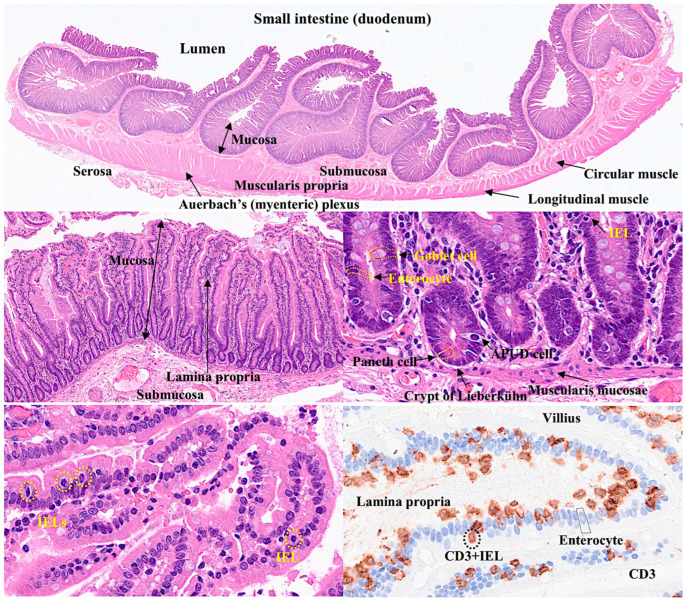
Histology of the small intestine. The small intestine comprises several layers, including the mucosa, submucosa, muscularis propria, and serosa. In the mucosa, intraepithelial lymphocytes (IELs) are found between epithelial cells (enterocytes). Using CD3 immunostaining, IELs can be easily identified under an optical microscope. Hematoxylin & eosin (H&E) staining at different microscope magnifications (original magnifications 50×, 100×, and 400×; (E), immunohistochemistry for CD3.

The pathogenesis of celiac disease is multifactorial. The pathogenesis includes a genomic background with the presence of several genetic factors [86] such as the close association with the HLA-DR3-DQ2 and/or DR4-DQ8 gene locus, which is highly present in patients with celiac disease [87]. Other gene loci related to metabolism and the immune system have also been identified using genome-wide association studies (GWASs), such as 3p21.31 (*CCR3* and *CCR2*), 4p27 (*KIAA1109*, *ADAD1*, *IL2*, and *IL21*), 6q15 (*BACH2*), 6q25.3 (*TAGAP*), 1q24.3 (*FASLG*, *TNFSF18*, and *TNFSF4*), 6q22.31 (*NKAIN2*), 10p15.1 (*PFKFB3* and *PRKCQ*), and 17q21.32 (*HOXB9*) [88]. Genome-wide gene expression studies have also highlighted similar biomarkers, including *APOC3*, *CYP3A4*, *OCLN*, *MAD2L1*, *MKI67*, *CXCL11*, and *IL17A* [89]. Figure 1 shows the histology of CD.

**Figure 2:**
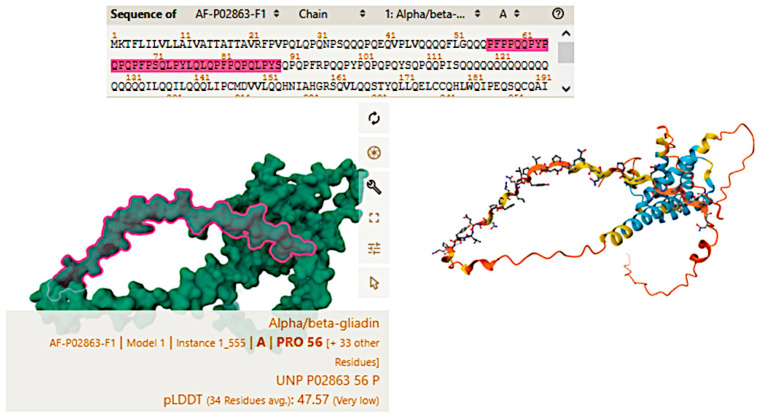
Alpha/beta-gliadin. Three-dimensional structure of gliadin of triticum aestivum (wheat). Amminoacids 56 to 89 are highlighted in the model. Uniprot reference: P02863. Last updated in AlphaFold DB version 2022-11-01.

Celiac disease is characterized by an abnormal mucosal immune response to gliadin fractions, resulting in chronic inflammatory infiltration of the lamina propria and epithelium and villous atrophy [90]. Regarding the adaptive immune response, the key factors are peptide 56-89 (α-gliadin) (Figure 2), which is resistant to gastrointestinal peptidases [91,92], tissue transglutaminase, and gliadin-reactive T cells.

**Figure 3:**
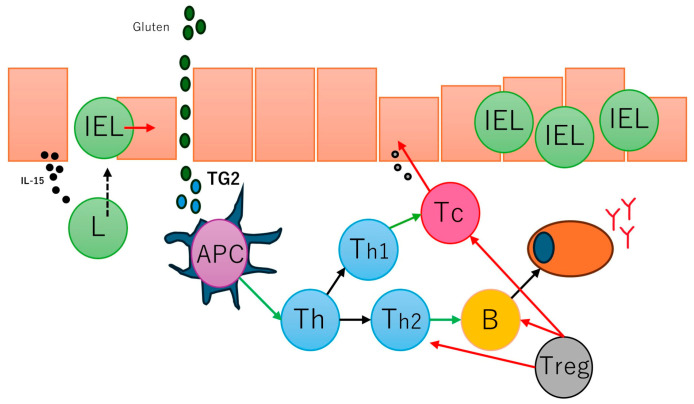
Simplified pathogenesis of celiac disease. The pathogenesis of celiac disease is multifactorial and involves several factors, including dietary gluten (gliadin), genetics (human leukocyte antigen (HLA)-DQ2/8 and other metabolic and immune-related non-HLA regions), environmental (dysbiosis, smoking), and immune factors. The immune factors include gluten-specific T-cell responses, autoantibody generation, cytokine generation, IELs cytotoxic transformation, and innate immune activation with epithelial stress [77]. APC, antigen-presenting cell; B, B lymphocyte; IEL, intra-epithelial lymphocyte; L, lymphocyte; Th, T helper cell; Tc, cytotoxic T lymphocyte; Treg, regulatory T lymphocytes; TG2, tissue transglutaminase 2.

In active and gluten-sensitive celiac disease, the number of intraepithelial lymphocytes increases, and these cells express interferon gamma and IL-10 [93]. The gammadelta T-cell receptor (γδTCR) is also found to be increased in intraepithelial lymphocytes [94] in addition to the common alfa-beta T-cell receptor (αβTCR); in a case of refractive celiac disease, intraepithelial lymphocytes may have an aberrant phenotype and restricted gene rearrangement [95,96]. Several antibodies are found in the serum of patients with celiac disease, including anti-gliadin antibodies (anti-AGAs), anti-deamidated gliadin peptide antibodies (anti-DGPs), anti-transglutaminase 2 antibodies (anti-TG2s), anti-R1-type reticulin antibodies (anti-ARAs), and anti-endomysia antibodies (anti-EMAs) [97]. Gluten peptides also activate innate immune responses, such as IL-15, intraepithelial lymphocytes, type 1 interferon (gamma), macrophages, monocytes, and dendritic cells, and induce dysbiosis [98]. A summary of the pathogenesis of celiac disease has been presented in our previous publications [77,78] (Figure 3).

Table 3 summarizes the epidemiology, pathogenesis, and clinical manifestations of celiac disease in adults.

The diagnosis of celiac disease includes a serologic evaluation, endoscopy with small bowel biopsy (duodenum), and HLA testing in selected patients [146,147]. The diagnostic approach of celiac disease depends on the individual’s disease probability. Individuals with low celiac disease probability should undergo serologic testing and, if positive, endoscopy and biopsy. When there is a high probability (highly suggestive clinical presentation and presence of risk factors), these patients should undergo both serologic testing and biopsy. Tissue transglutaminase (tTG)-IgA antibody is the single preferred test for the detection of celiac disease in adults. Serum antibody assays include autoantibodies, antigliadin, anti-endomysia, and anti-tissue transglutaminase [97,146,147,148].

The endoscopic characteristics of celiac disease have low sensitivity and include atrophic mucosa with loss of folds, fissures, nodularity, and prominent submucosal vascularity [149,150].

The histological features of celiac disease range from mild alteration with increased IELs to severe atrophy with loss of villi, high epithelial apoptosis, and crypt hyperplasia.

Table 4 presents the histological classification based on Marsh [151].

The definition of celiac disease is a condition in which a chronic inflammation of the mucosa improves morphologically in a gluten-free diet and relapses when it is reintroduced. Therefore, the treatment consists of lifelong adherence to a gluten-free diet and identification and treatment of nutritional deficiencies. Investigational approaches include the use of transglutaminase inhibitors [152,153,154,155].

### 1.4. LAIR1

Leukocyte-associated immunoglobulin-like receptor 1 (LAIR1), also known as CD305, is an immune-inhibitory receptor found on mature hematopoietic cells, particularly on immune cells such as mononuclear cells, natural killer cells, and T- and B-lymphocytes [156].

**Figure 4:**
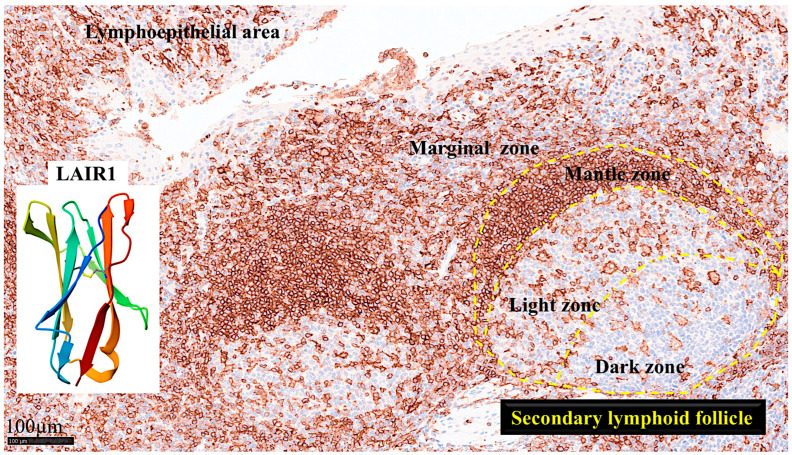
LAIR1 expression in reactive tonsils and human LAIR1 crystal structure. Immunohistochemistry staining of LAIR1 in reactive tonsil controls from human samples. LAIR1 was expressed in both follicles and interfollicular areas of reactive tonsils. In the secondary follicles, the pattern was characteristic of macrophage/dendritic cells in the germinal center and of naïve B-lymphocytes in the mantle zone. In the interfollicular area, the pattern was compatible with dendritic cells, including in the lymphoepithelial area. Because LAIR1 + IELs were found, this research study aimed to characterize LAIR1 expression in IELs of the intestinal mucosa, with a focus on the small intestine, and celiac disease as a pathological counterpart. The crystal structure of human LAIR1 in the C2 space group is also shown; experimental data were obtained using the X-ray diffraction method (https://www.rcsb.org/structure/3RP1; accessed on 25 March 2025). Tissue: human reactive tonsil (Department of Pathology, Tokai University Hospital). Scale bar: 100 micrometers.

The gene is located in the 19q13.4 region and is known as the leukocyte receptor cluster, which contains several genes that encode leukocyte receptors of the immunoglobulin superfamily. LAIR1 induces cell death and inhibits cytokine release and the activation of the NFKB pathway in myeloid leukemia [157,158,159]. Figure 4 shows the structure of LAIR1. It is a type I glycoprotein comprising 287 amino acids belonging to the family IR [160].

A comprehensive review of the role of LAIR1 in immune cell responses and neoplasia was recently performed by Poggi A. et al. [161], and the association with immune disorders and hematological neoplasms was reported by Van Laethem F. et al. [156]. Immune cell function can be modulated using inhibitory receptors. Many of these inhibitory receptors recognize a limited number of specific ligands. However, a subgroup of inhibitory receptors, called inhibitory pattern recognition receptors (iPRRs) can bind a large number of ligands of structural similarity [162,163]. LAIR1 belongs to the iPRR group and recognizes common structural patterns in collagens and collagen domain-containing proteins [164].

Autoimmune diseases are characterized by a pathological response to self- or autoantigens. These disorders can be either systemic (such as systemic lupus erythematosus and vasculitis) or organ-specific (such as autoimmune thyroiditis and multiple sclerosis) and can be either acute or chronic [165]. The pathogenesis of autoimmune diseases is complex [165] and involves breakdown or defects in immune tolerance, defects in active regulation and control of autoreactivity (FOXP3 + Tregs, IL-10, CTLA-4, TGF-beta) [166], defects in regulation of autoimmune B-cell responses (autoreactive B cells) [167], targeting of cell surface and soluble antigens and immune complex formation, immune complexes (between autoantibodies and the corresponding autoantigen present in the circulation and/or on cell surfaces) [168], effector T-cell-mediated injury (cytotoxic cells) [169], innate immune mechanisms (pattern recognition receptors) [170], specific T-cell subsets (Th1, Th2, Th17, Tregs), cytokines, internalization of autoantibodies (myopathies), and dysregulation of apoptosis (autoimmune lymphoproliferative syndrome (ALPS) [171], systemic lupus erythematosus, rheumatoid arthritis, systemic sclerosis) [172]. Regarding ulcerative colitis, we recently described steroid-requiring patients with high infiltration of LAIR1+ cells in the lamina propria [173].

LAIR1 has been associated with the pathogenesis of several autoimmune diseases, including systemic lupus erythematosus (SLE) [174], rheumatoid arthritis (RA) [175], allergy (airway hyper-reactivity and asthma) [176], graft rejection (kidney transplant) [177], and chronic hepatitis [178]. Regarding neoplasia of the hematopoietic and lymphoid system, we have recently described the role of LAIR1 in the pathogenesis of follicular lymphoma [179] and diffuse large B-cell lymphoma [180].

Epidemiological studies continue to identify celiac disease-associated diseases such as inflammatory arthritis and irritable bowel disease [181]. Celiac disease is a systemic immunological disorder caused by gluten (gliadin and other prolamin) in genetically predisposed individuals. The pathogenesis is complicated and remains a subject of research. The basic treatment is elimination of products that may contain gluten from the diet. However, new therapeutic strategies are being developed, such as supplementation with exogenous endopeptidases, immune response modification, and the use of zonulin and transglutaminase 2 inhibitors [182]. Due to the relevance of LAIR1 immune-inhibitory receptor on the function and activation of mature hematopoietic cells, particularly on immune cells such as mononuclear cells, natural killer cells, and T and B-lymphocytes, the role of LAIR1 in intestinal mucosa (IELs) and celiac disease pathogenesis warrants analysis.

### 1.5. Aim of the Study

This study aimed to analyze the phenotype of intraepithelial lymphocytes (IELs) and the lamina propria in the small intestine, including LAIR1, and to confirm the expression of LAIR1 in celiac disease.

The highlights are as follows:

- In the small intestine control, IELs exhibited a cytotoxic T-cell phenotype and were positive for CD3, CD8, CD103, TCRβ, and LAIR1.
- CD was characterized by higher LAIR1-positive IELs and LP immune cells than the small intestine control (*p* = 0.004).
- Higher intestinal lesions evaluated by Marsh scoring were correlated with higher LAIR1 (*p* < 0.001).
- CD was characterized by gene-set enrichment of the LAIR1 pathway using an independent transcriptomic dataset.

## 2. Materials and Methods

### 2.1. Patients and Samples

This study included 16 cases of celiac disease (total number of biopsies n = 57), 18 cases of small intestine control, 11 reactive lymphoid tissue, and 3 lymphoma cases (used as immunohistochemical internal control). The celiac cases were selected from the Department of Pathology of Hospital Clinic Barcelona, Spain, as described in our previous publications [77,78]. The patients were diagnosed with celiac disease following the conventional diagnosis, with clinical criteria, positive celiac serology, and histological criteria, including the presence of increased intraepithelial lymphocytes with crypt hyperplasia (Marsh type 2) or villous atrophy (Marsh type 3). The detailed data are presented in Appendix A, Table A1.

This study was conducted according to the principles of the Declaration of Helsinki for human experimentation. Ethical Committee of Tokai University approved this study (IRB14R-080 and IRB20-156).

### 2.2. Immunohistochemistry

Several immunohistochemical markers were analyzed in the tissue samples using a Leica Bond Max automated stainer according to the manufacturer’s instructions. The primary antibodies that were used were the following: CD3 (clone LN10, Leica Biosystems, Leica K.K., Shinjuku-ku, Tokyo, Japan), CD4 (4B12, Leica), CD8 (4B11, Leica), CD103 (EP206, Leica), granzyme B (11F1, Leica), TCRβ (TRBC1/TCRβ constant region 1 (E6Z3S) Rabbit mAb #79485, Cell Signaling Technology K.K., Chiyoda-ku, Tokyo, Japan), TCRδ (TRDC/TCRδ (E2E9T) XP^®^ Rabbit mAb #55750, Cell Signaling), CD56 (CD56-504-L-CE, Leica), CD16 (CD16-L-CE, Leica), LAIR1 (CD305, JAVI82A, created by Giovanna Roncador, Spanish National Cancer Research Center (CNIO, Centro Nacional de Investigaciones Oncologicas, calle Melchor Fernandez Almagro, 3, 28029 Madrid, Spain), PD-L1 (73-10, Leica), PD1 (CD279, NAT105, CNIO), BTLA (CD272, FLO67B, CNIO), TOX2 (TOM924D, CNIO), HVEM (TNFRSF14, ab47677, Abcam Tokyo, Ota city, Tokyo, Japan), CD163 (CD163-L-CE, Leica), HLA-DP-DQ (JS76, CNIO), IL4I1 (BALI265E,543H,573B, CNIO), and FOXP3 (236A, CNIO). The details of the primary antibody details are presented in Table 5.

Confocal microscopy was performed as described previously [183] using a Fluoview FV3000 confocal laser scanning microscope (Olympus K.K, Hachioji, Tokyo 192-8507, Japan) with Alexa Fluor 488 and 594 and DAPI dyes.

The immunohistochemical expression of BTLA in celiac disease was imported from our previous publication, including the histological slides, and all data reanalyzed [77].

LAIR1 was evaluated in a semiquantitative manner as low (1+, <20%), intermediate (2+, 20–50%), and high (3+, >50%).

### 2.3. Image Classification

Image classification based on the immunohistochemical expression of LAIR1 was performed using transfer learning and the ResNet18 deep learning model, as recently described [77,184,185]. All histological slides were scanned using a NanoZoomer S360 digital slide scanner (Hamamatsu Photonics K.K., Hamamatsu City, Shizuoka Pref., 430-8587, Japan). After visualization using the NDP.view2 image viewing software U12388-01 (Hamamatsu Photonics K.K.), the whole-tissue sections were exported into a jpeg file at 200× magnification and 150 dpi. The images were split into images patches of 224 × 224 × 3 using PhotoScape v3.7 (MOOII Tech, Korea; http://www.photoscape.org/; last accessed on 13 October 2025). All image patches were manually revised to exclude artifacts such as broken, folded, and nondiagnostic images. Image patches that were not 224 × 224 in size, those without tissue, or those with less than 20% tissue were automatically discarded. The image patches were pooled into 3 different folders, and the data were split into 3 sets: training set (70%) for training the network, validation set (10%) for testing its performance during training, and test set (20%) used after training to assess how well the network performed on new data. Grad-CAM analysis was used as an explainable AI method to visualize which areas of the input image were most important for model prediction and image classification. The methodology was performed as previously described in our previous publications [78,173,184]. All analyses were performed using a desktop equipped with an AMD Ryzen 9 5900X 12-Core Processor 3.70 GHz (Advanced Micro Devices, Inc., Osaki, Shinagawa-ku, Tokyo 141-0032, Japan), 48.0 GB of RAM, an NVIDIA GeForce RTX 4080 SUPER (16 GB) GPU (NVIDIA, Santa Clara, CA 95051, USA), and MATLAB R2023b Update 10 (23.2.0.2859533) 64-bit (win64) 27 January 2025 (MathWorks, Natick, MA 01760-2098, USA).

In the confusion matrix, the image patches were recorded as true positive (TP), false positive (FP), false negative (FN), and true negative (TN). The accuracy performance parameter was calculated as follows: accuracy = (TP + TN)/(TP + TN + FP + FN).

The relevant code functions were as follows: [imdsTrain, imdsVal, imdsTest] = splitEachLabel(imds, 0.7, 0.1, “randomized”); imdsTrain = shuffle(imdsTrain); YPred = classify(trainedNetwork_1, imdsTestAug); accuracy = sum(YPred == imdsTest.Labels)/length(YPred); scores = predict(trainedNetwork_1, imdsTestAug); confusionchart(YPred, imdsTest.Labels); wronglyPredicted = find(YPred~= imdsTest.Labels); imdsTest.Files{wronglyPredicted}

### 2.4. Gene Expression Analysis

A suitable independent series of celiac disease was searched in the Gene Expression Omnibus database, and the public dataset published by Dr Worf J et al. [186] was selected. This dataset includes transcriptome analysis of 48 duodenal biopsies of 26 children and adolescents diagnosed with celiac disease and 22 children without celiac disease as controls. Frozen tissue biopsies were obtained, and total RNA was extracted using a Qiagen AllPrep^®^ DNA/RNA Microkit (Qiagen Inc., Santa Clarita, CA 91355, USA). Gene expression was assessed using the Illumina HumanHT-12 v4.0 beadchip [186] (Illumina Inc., Sand Diego, CA 92122 USA).

Gene set enrichment analysis (GSEA) was performed using GSEA software version 4.4.0 (build 18) from Broad Institute, Inc. (Merkin Building, 415 Main St., Cambridge, MA 02142, USA). The analysis procedure was as previously described [77]. STRING version 12.0 (SIB, Swiss Institute of Bioinformatics; CPR, Novo Nordisk Foundation Center Protein Research; University of Copenhagen, Blegdamsvej 3B, DK-2200 Copenhagen N, Denmark); and EMBL, European Molecular Biology Laboratory; EMBL Hamburg, c/o DESY, Building 25A Notkestraße 8522607 Hamburg, Germany) were used for the functional network association analysis [187].

### 2.5. Statistical Analyses

All statistical analyses were performed using IBM SPSS version 27.0.1.0 (64-bit edition; IBM Corporation, Armonk, New York, NY, USA). Comparison between groups was performed using crosstabulation and chi-square test and the Mann–Whitney U nonparametric test. Spearman’s rho test was used to determine nonparametric correlations between genes. *p*-values ≤ 0.05 were considered statistically significant.

## 3. Results

### 3.1. Immunophenotype of IELs in Intestinal Mucosa Control

IELs were defined as lymphoid cells within the mucosal epithelial layer. CD3 staining was used as a reference. Under control physiological conditions, IELs were characterized by a T-cell phenotype that is positive for CD3 and a cytotoxic phenotype with CD8 expression. Cytotoxic granules were occasionally identified by granzyme B staining. Most IELs were positive for CD103/ITGAE. Most IELs expressed TCRβ chains; therefore, they expressed the TCRαβ chains. CD56 + IELs, TCRδ chain + IELs (i.e., TCRγδ + IELs), and TOX2 + IELs were occasionally found. Notably, all IELs were LAIR1-positive by immunohistochemistry.

**Figure 5:**
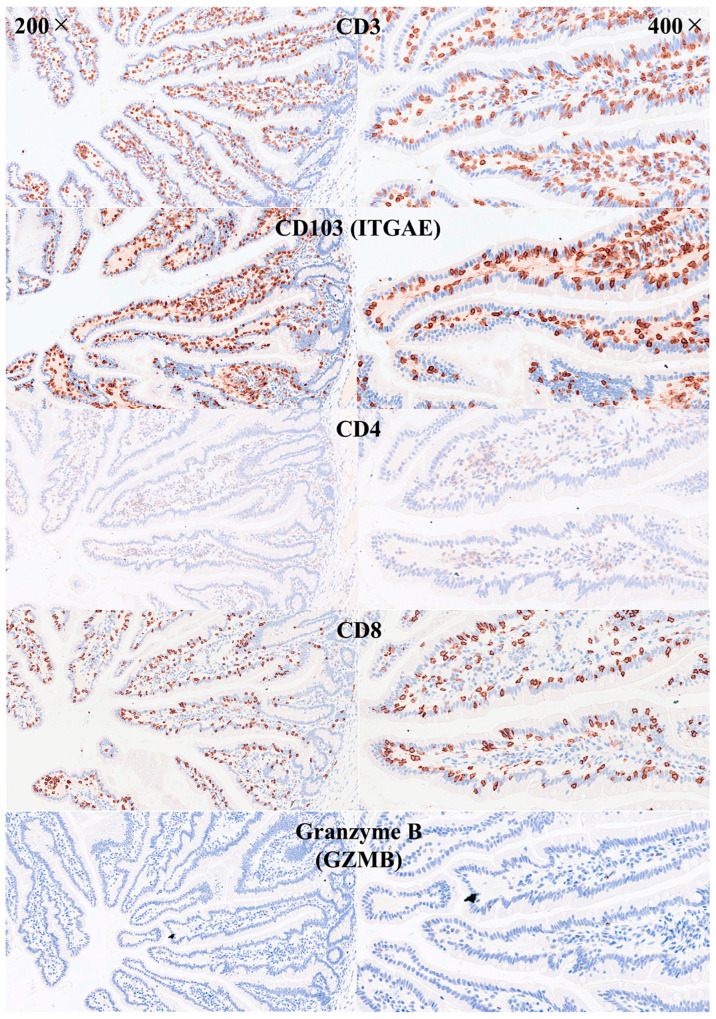
Immunophenotype characterization of IELs in the intestinal mucosa control. Most IELs were CD3+, CD103+, and CD8+. CD4+ cells were mainly found in the lamina propria. Original magnification 200× and 400×.

**Figure 6:**
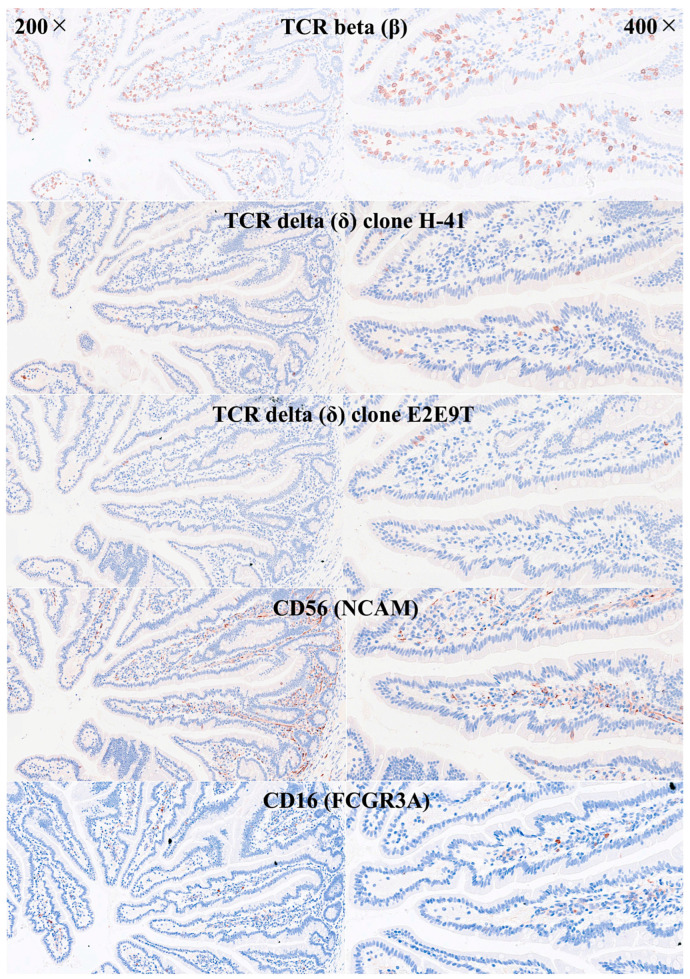
Immunophenotype characterization of IELs in the intestinal mucosa control. Most IELs expressed TCRβ chains. Occasionally, CD56 + IELs were found, as well as TCRδ chain-positive IELs (i.e., TCRγδ + IELs). TCRB, TCR beta (β); TCRD, TCR delta (δ). TCR delta was analyzed using two different clones: H-41 (Santa Cruz, sc-100289) and E2E9T (Cell Signaling, TRDC/TCRδ (E2E9T) XP® Rabbit mAb #55750). Original magnification 200× and 400×.

**Figure 7:**
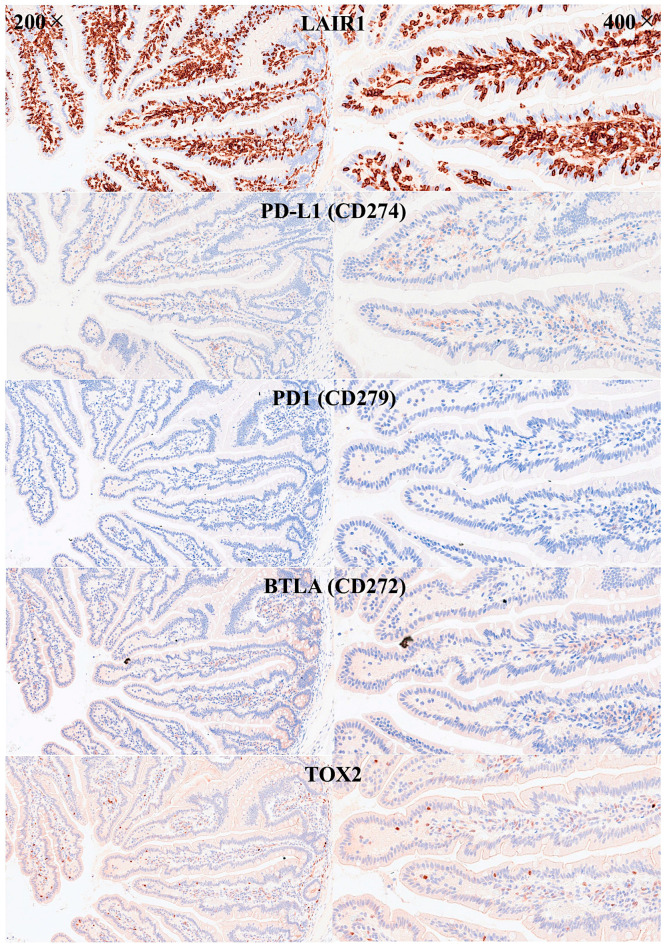
Immunophenotype characterization of IELs in the intestinal mucosa control. IELs were diffusely and strongly positive for LAIR1. LAIR1 also marked the inflammatory infiltrate of the lamina propria. The expression of PD-L1 and BTLA was limited in the lamina propria. Occasional PD1+ cells were identified, and TOX2+ IELs were occasionally found. Original magnification 200× and 400×.

**Figure 8:**
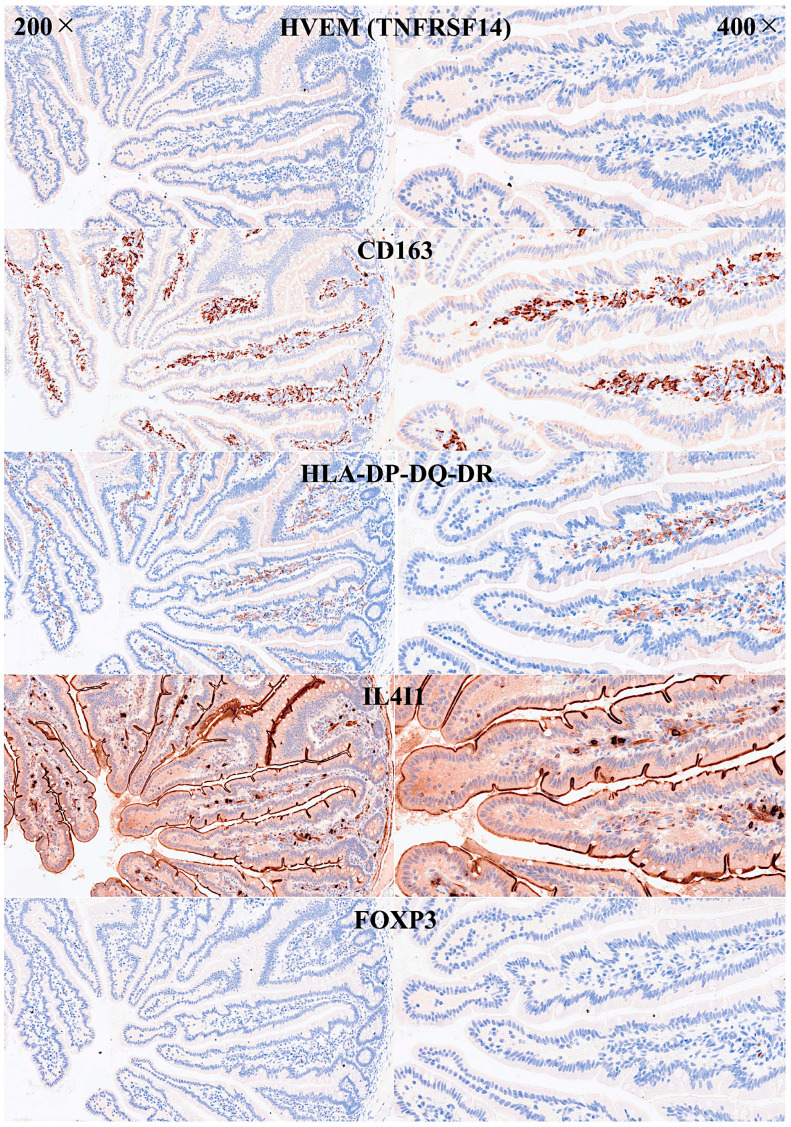
Immunophenotype characterization of IELs in intestinal mucosa control. Antigen-presenting cells (APCs), mainly macrophages and dendritic cells, were identified using CD163 and HLA-DP-DQ-DR in the lamina propria. Few FOXP3 + Tregs were identified in the lamina propria. IL4I1, Interleukin 4 Induced 1.

**Figure 9:**
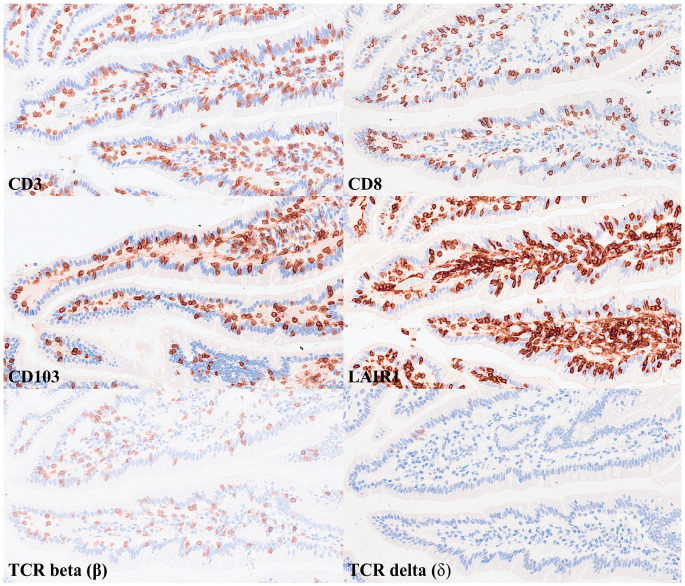
Main phenotype of IELs in control intestinal mucosa. This figure summarizes the main immunophenotypes of IELs, including CD3+, CD8+, CD103+, LAIR+, and TCRβ+. An area with marked aggregation of IELs and immune cells in the lamina propria is shown. Original magnification 400×.

**Figure 10:**
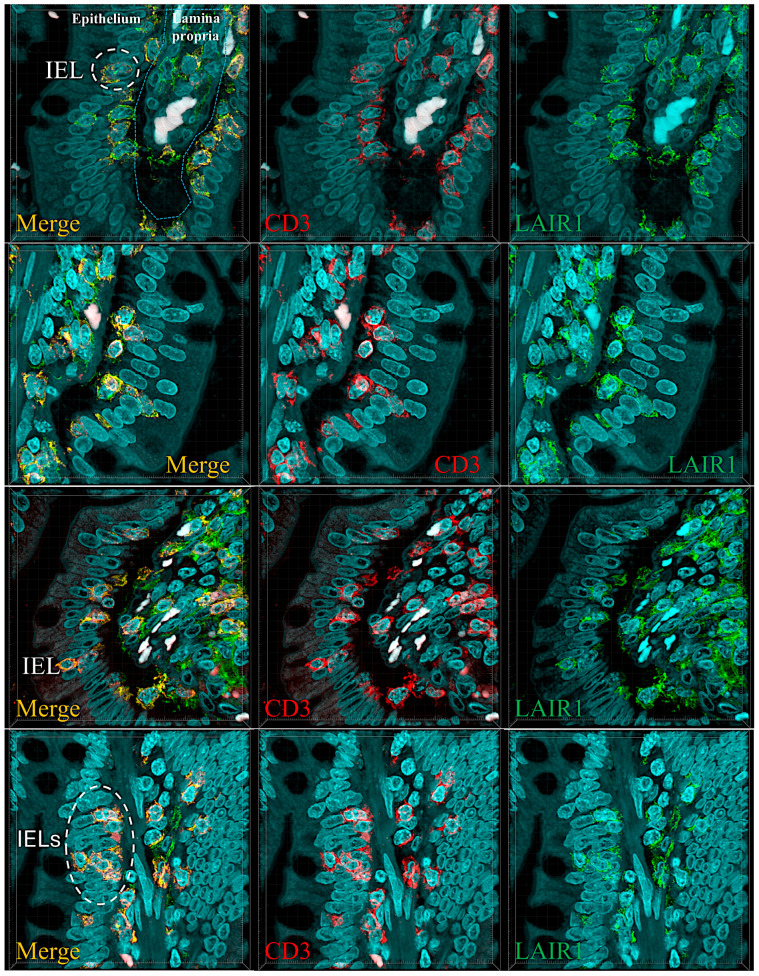
Confocal microscopy showing double immunofluorescence between CD3 (red) and LAIR1 (green) in the control small intestine. The IELs were double-positive for CD3 and LAIR1. Original magnification 600×.

**Figure 11:**
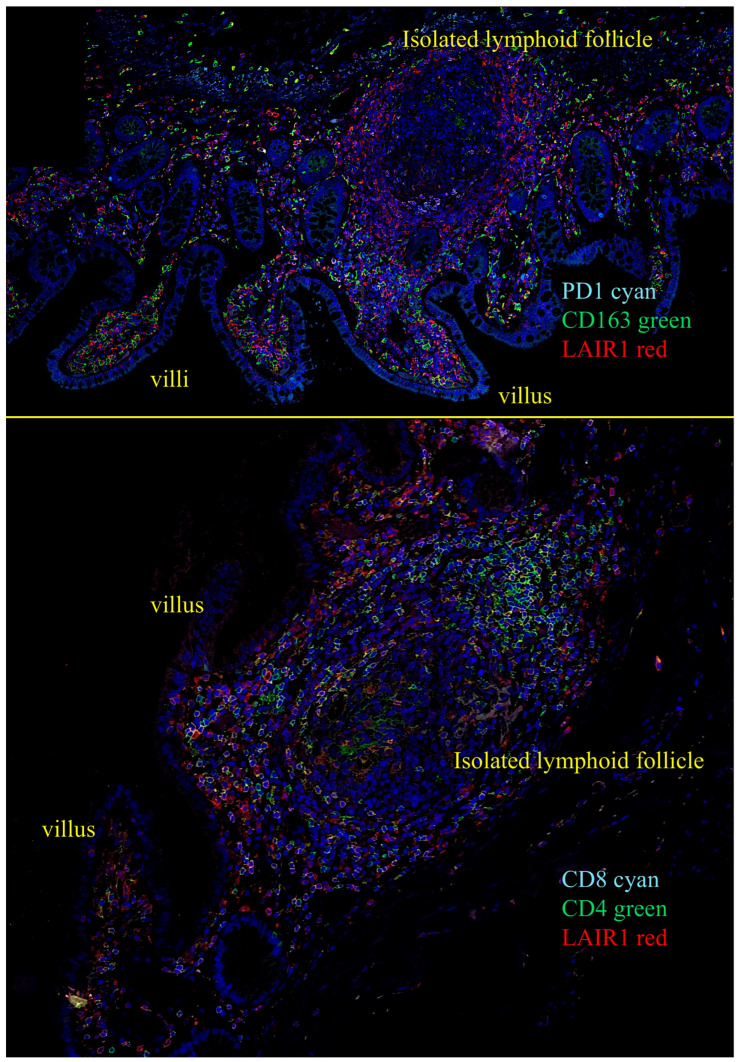
Multicolor immunofluorescence of LAIR1 in relation to other immune markers in human intestinal control. Original magnification 600×.

**Figure 12:**
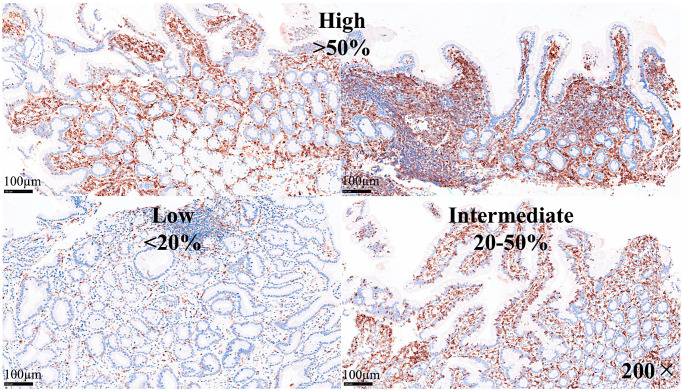
Expression of LAIR1 in celiac disease. LAIR1 expression in the lamina propria ranged from low (1+, <20%, 1/16, 6.3%), intermediate (2+, 20–50%, 8/16, 50%), and high (3+, >50%, 7/16, 43.8%). Higher histological alterations correlated with higher values in the Marsh classification (*p* < 0.001).

Abundant CD163 + macrophages/dendritic cells were found in the lamina propria, which also expressed HLA-DP-DQ. CD4 + cells were mainly found in the lamina propria; however, clusters attached below the epithelial basal membrane were found. BTLA + cells were found in the lamina propria, which is consistent with our previous results [77]. PD-L1 expression was limited to the lamina propria in a pattern compatible with APC (macrophages, dendritic cells). Regulatory T-lymphocytes were identified in the lamina propria using the FOXP3 marker. Table 4 summarizes the findings. Characteristic images are shown in Figure 4, Figure 5, Figure 6 and Figure 7. Figure 8 confirms that the IELs are CD3 and LAIR1 double-positive using confocal microscopy. Notably, LAIR1 staining revealed that many cells of the lamina propria were LAIR1-positive (Figure 5, Figure 6, Figure 7, Figure 8, Figure 9, Figure 10, Figure 11 and Figure 12) (Table 6).

### 3.2. Multicolor Analysis of LAIR1 and Other Immune Markers

We performed quadruple and triple immunofluorescence analyses using confocal microscopy. The combinations that also included nuclear staining were as follows: PD1 (cyan), CD163 (green), and LAIR1 (red); and CD4 (green), CD8 (cyan), and LAIR1 (red).

In the human mucosa, CD4-positive, and probably, CD8-positive cells, were positive for LAIR1. PD1-positive cells also expressed LAIR1. CD163-positive cells (M2-like macrophages) were partially positive for LAIR1 in the interfollicular area and/or lamina propria. Mantle zone B-lymphocytes were LAIR1-positive but not in the germinal centers (Figure 11).

### 3.3. Analysis of LAIR1 Expression in Patients with Celiac Disease

Table 7 shows the distribution of cases according to the Marsh histological classification. In celiac disease, Marsh 0-2 accounted for 31.3%, and Marsh 3 for 68.7% of the cases. As expected, celiac disease cases had higher values in the Marsh classification (*p* < 0.001).

The protein expression of LAIR1 using immunohistochemistry was evaluated in the celiac disease biopsies. LAIR1 was expressed in the IELs but also in the inflammatory infiltrate of the lamina propria, and the distribution of LAIR1 + IELs was heterogeneous within and between biopsies.

LAIR1 expression in the mucosa control was low (1+) in 6/18 (33.3%) or intermediate (2+) in 12/18 (66.7%). In celiac disease, LAIR1 expression ranged from low (1+, 1/16, 6.3%), intermediate (2+, 8/16, 50%), and high (3+, 7/16, 43.8%). Therefore, celiac disease was characterized by higher LAIR1 expression (*p* = 0.004) (Table 8).

The histological features of celiac disease ranged from mild alteration with increased IELs to severe atrophy with loss of villi, high epithelial apoptosis, and crypt hyperplasia. Table 9 shows the correlation between Marsh histological classification and LAIR1. Higher histological lesions were correlated with higher LAIR1 expression (*p* < 0.001).

### 3.4. Image Classification of Celiac Disease, Small Intestine Control, and Reactive Tonsil Control Based on LAIR1 Immunohistochemical Expression

**Figure 13:**
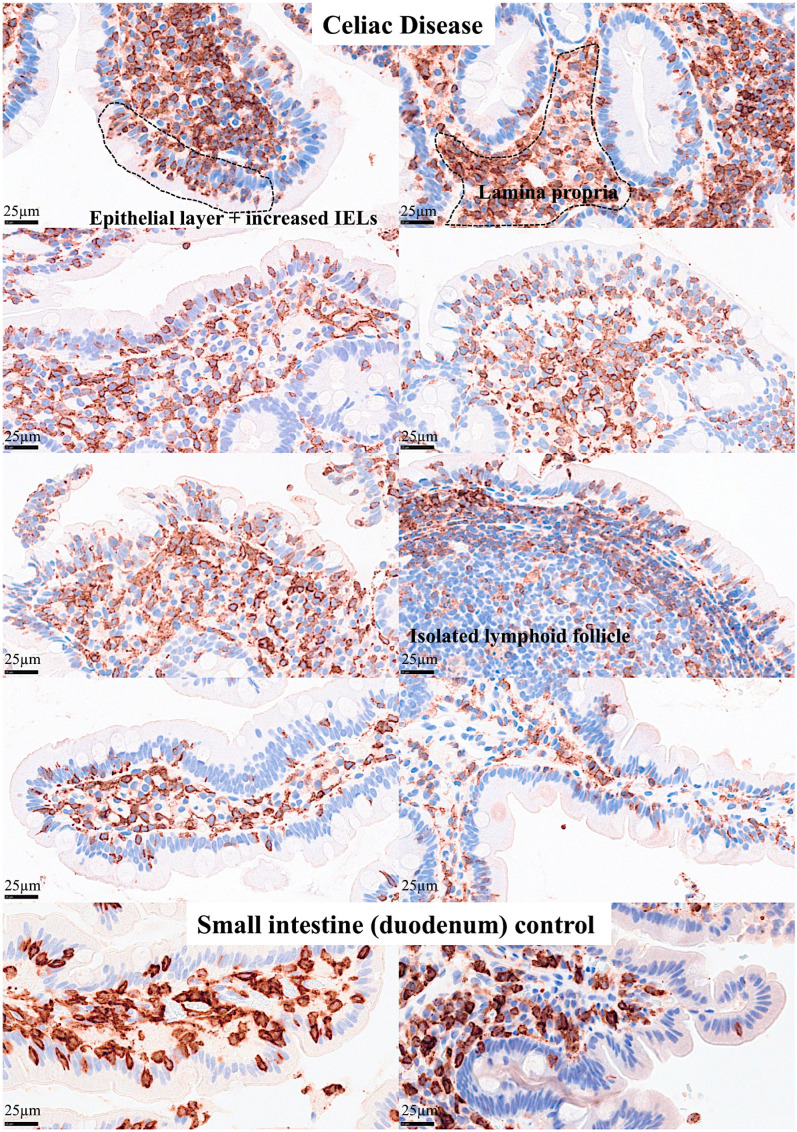
Additional LAIR1 images. Additional images of celiac disease and small intestine control (duodenum) stained with LAIR1 marker are shown. In the small intestine control, LAIR1 expression was limited to the lamina propria and in IELs when present. In celiac disease, the infiltration of LAIR1+ cells in the lamina propria was variable but was high (3+) or intermediate (2+) in most cases. Increased numbers of LAIR1+ IELs were found in the epithelial layer of celiac disease cases. In addition to immune system cells, celiac disease showed architectural changes such as villus atrophy and crypt hyperplasia. Original magnification: 800×.

Images of LAIR1 protein expression analyzed by immunohistochemistry in celiac disease, small intestine control, and reactive tonsils were used as input data in a ResNet18 model [185] (Figure 13).

The ResNet18 model comprises 18 layers, including convolutional layers and residual blocks [188,189,190]. The series included 11,367 image patches of celiac disease, 11,630 of the small intestine control, and 8147 of the reactive tonsil control. The image patches were pooled into three different folders, and the data were split into three sets: training set (70%) for training the network, validation set (10%) for testing its performance during training, and test set (20%) used after training to assess how well the network performed on new data.

**Figure 14:**
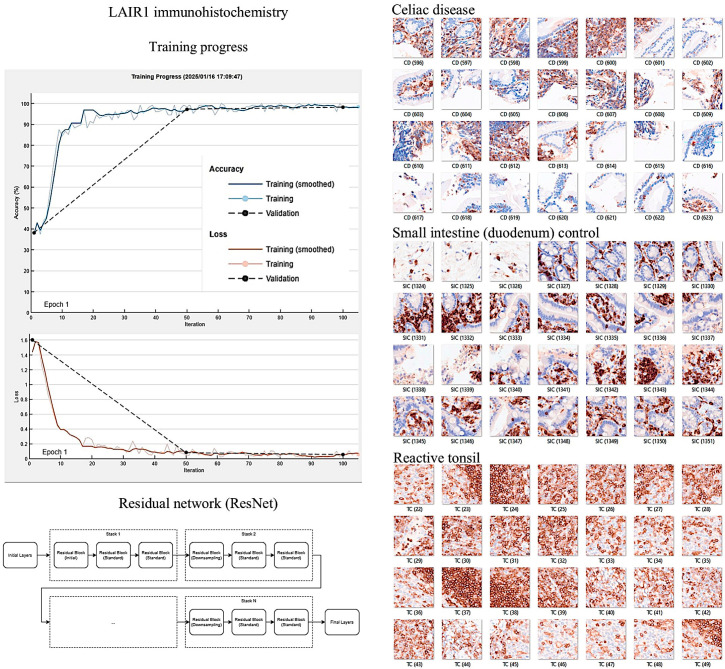
Histological image classification. A convolutional neural network based on the ResNet18 model was used to classify LAIR1 immunohistochemical images of celiac disease, small intestine control (duodenum), and reactive tonsil. Residual networks (ResNets) are a type of deep network consisting of building blocks with residual (skip or shortcut) connections. These connections allow the input to skip the main branch’s convolutional units, thus providing a simpler path through the network. By allowing the parameter gradients to flow more easily from the final layers to the earlier layers of the network, residual connections mitigate the problem of vanishing gradients during early training. After 5 epochs in the training, the validation accuracy was 99.5%. After image patch classification using the test (holdout) series, the accuracy was 99.6%. Original magnification 200×.

**Figure 15:**
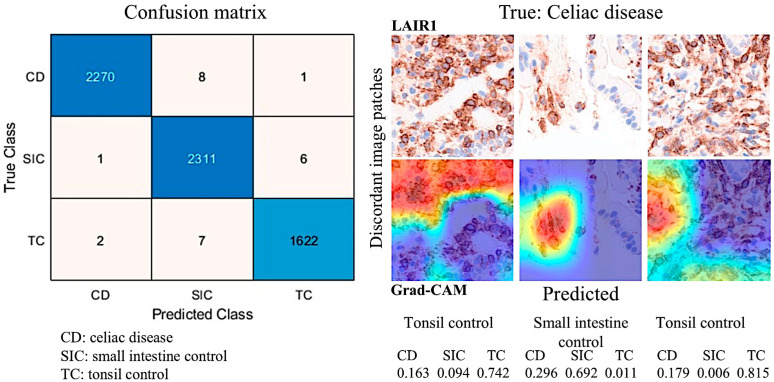
Image classification using the LAIR1 image patches. A ResNet18 model was created to classify histochemical images of celiac disease (CD), small intestine control (SIC), and reactive tonsil control (TC). The image classification results in the test set are summarized in a confusion matrix. The confusion matrix shows how the accuracy was 99.6% after image patch classification using the test (holdout) series. Grad-CAM technique was used to understand why the deep learning network made its classification decisions in incorrectly classified cases. For each of the 3 examples, the class probability is shown. Original magnification 200×.

After five epochs in the training, the validation accuracy was 99.5%. After image patch classification using the test (holdout) series, the accuracy was 99.6%. The confusion matrix shows the distribution of image patches, including correctly and misclassified patches (Supplementary Data). The Grad-CAM technique was used to understand why the deep learning network made its classification decisions in incorrectly classified cases (Figure 14 and Figure 15).

### 3.5. Analysis of LAIR1 in Celiac Disease Using Gene Expression Data

**Figure 16:**
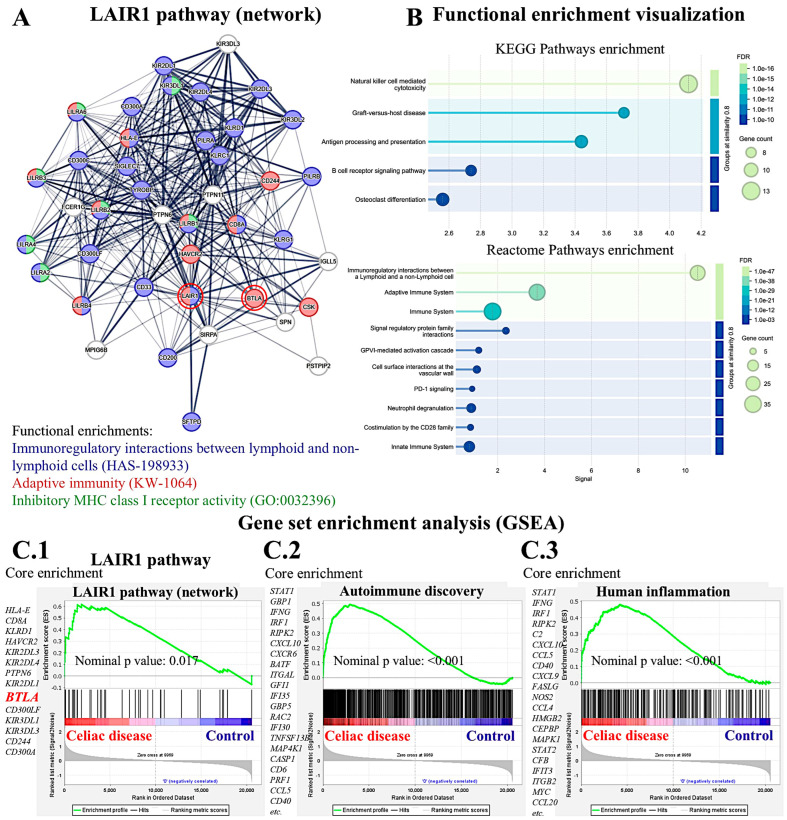
Functional network and gene set enrichment analysis (GSEA). The LAIR1 network and pathway were analyzed using functional network association analysis and GSEA. The analysis highlighted the importance of LAIR1 and its partners in immune regulation (**A**). Functional enrichment analysis confirmed these findings using KEGG and Reactome pathways (**B**). GSEA confirmed the enrichment (overexpression) of the LAIR1 pathway in celiac disease patients (**C.1**), as well as other autoimmune (**C.2**) and human inflammation genes (**C.3**). Of note, BTLA was highlighted both in the network and GSEA analysis. Note: 1.0 × 10^−16^ equals to 1.0e-16.

A suitable independent series of celiac disease was used to validate the findings of LAIR1 at the gene expression level. A functional network association analysis approach was used to define the LAIR1 pathway. The result of the network analysis is shown in Figure 16A: in the network (i.e., the LAIR1 pathway), the partners are shown and are classified in different colors according to their immunoregulatory interactions, adaptive immunity, and MHC class I inhibitory function. Functional enrichment visualization confirmed the immunoregulatory function of LAIR1 and its partners (Figure 16B), and the association (i.e., enrichment, upregulation) of the *LAIR1* pathway in patients with celiac disease was confirmed in the gene set enrichment analysis (GSEA) (Figure 16(C.1)). The enrichment of autoimmune and human inflammation-associated genes was also confirmed by GSEA (Figure 16(C.2,C.3)). Of note, both network analysis and GSEA highlighted the *BTLA* marker (Figure 16(A,C.1)). BTLA was close to LAIR1 in the network, and in the GSEA, BTLA was found within the core enrichment.

**Figure 17:**
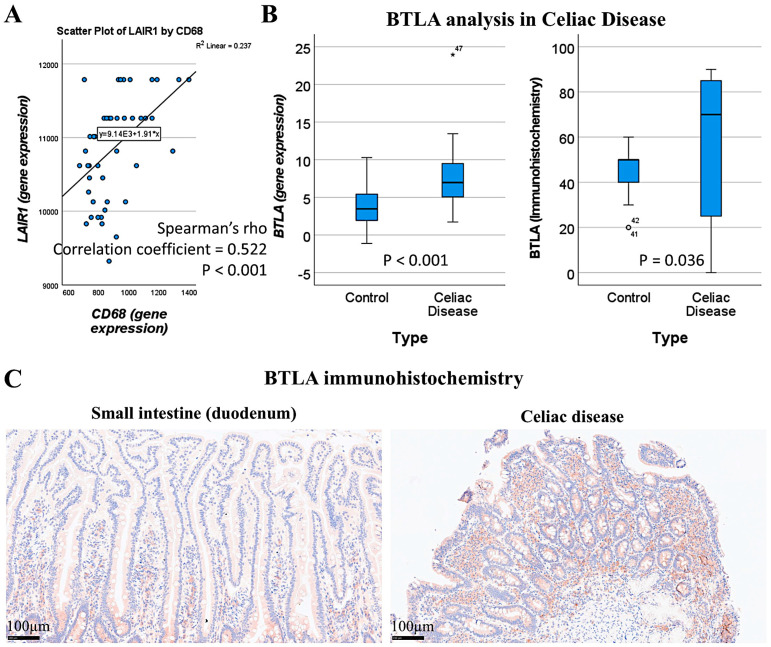
Correlation between LAIR1 and CD68+ macrophages, on the one hand, and BTLA analysis. By gene expression, LAIR1 correlated with CD68 pan-macrophage marker, as shown in the immunofluorescence confocal analysis (**A**). LAIR1 network and GSEA analysis pinpointed BTLA marker, which is another immune co-inhibitory marker. BTLA was confirmed to be overexpressed both at RNA and protein levels in patients with celiac disease (**B**). An example of the different expression of BTLA in the mucosa of control and celiac disease is shown (**C**). ^○^, outlier; *, extreme outlier. Note: 1.0 × 10^3^ equals to 1.0E+3.

Our study results showed that LAIR1 identified CD163+ cells in the lamina propria (Section 3.2, Figure 10). By gene expression, *LAIR1* correlated with CD68, a pan-macrophage marker (Figure 17A).

BTLA was further analyzed, and gene expression levels were confirmed to be overexpressed in celiac disease (*p* < 0.001) (Figure 17B). At the protein level in our series, high BTLA expression was confirmed in celiac disease cases (*p* = 0.036) (Figure 17B,C).

## 4. Discussion

Celiac disease is a gluten-sensitive enteropathy and common immune-mediated inflammatory condition of the small intestine caused by sensitivity to dietary gluten and related proteins in genetically predisposed individuals [191]. In Western countries, celiac disease is estimated to affect approximately 1% of the population. Celiac disease clinically presents as heterogeneous; therefore, it continues to be underestimated [191].

There are several phenotypes of celiac disease. Symptomatic diseases include classic and nonclassic celiac diseases. Classic celiac disease is a gluten-sensitive enteropathy characterized by diarrhea, signs and symptoms of malabsorption, villous atrophy, and resolution upon withdrawal from gluten-containing foods [79]. Nonclassic celiac disease is also known as “atypical”, and these patients lack the classic symptoms of malabsorption and only present with minor gastrointestinal complaints. However, duodenal biopsies show villous atrophy, the production of celiac autoantibodies, such as anti-tissue transglutaminase, and extraintestinal manifestations [123,192].

The other phenotypes include subclinical or asymptomatic, potential, latent, and refractory celiac disease. Refractory celiac disease is defined as the persistence of clinical symptoms and villous atrophy despite adherence to a gluten-free diet. Failure to improve on a gluten-free diet is mostly due to noncompliance. However, in few cases, a pure refractory condition is found: refractory celiac disease type 1 (normal population of IELs), the semi-malignant inflammatory condition (refractory type 2; aberrant immunophenotype and T-cell receptor clonality analysis of IELs), transformation to enteropathy-associated T-cell lymphoma (EATL), collagenous sprue, or alternative diagnosis of autoimmune enteropathy [126,127,193].

The cause of refractory disease is unknown, and immunosuppression has been the treatment of choice. Traditional glucocorticoids, such as intravenous hydrocortisone and oral prednisolone, are used. Alternative immunosuppressant therapies include azathioprine, 6-mercaptopurine, and thioguanine [126,194,195,196,197]. A monoclonal antibody therapy using anti-CD52 (alemtuzumab) was reported [198].

Badran YR et al. reported eight cases of immune checkpoint inhibitor-associated celiac disease, suggesting that the drugs disrupted gut immune homeostasis and the gut immune tolerance mechanism [199]. In that study, immunohistochemical analysis of several markers included CD3, CD8, TCRγδ, PD1, CD68, PD-L1, and quantification of IELs [199]. In our study, we analyzed several immuno-oncology markers in a small intestine control and, later, LAIR1 expression in celiac disease. Our findings showed that in the small intestine, LAIR1 expression is found not only in IELs but also in lamina propria immune cells. LAIR1 was diffusely expressed in celiac disease.

LAIR1 belongs to the family of immune-inhibitory receptors and is expressed by mature hematopoietic cells, particularly in natural killer (NK) and T/B-lymphocyte immune cells [198]. Beyond the physiological function of immune homeostasis and immune tolerance, LAIR1 has been involved in several autoimmune and inflammatory conditions and neoplasia [156], including allergy [200], systemic lupus erythematosus [201], rheumatoid arthritis [175,202,203], graft rejection [204], breast carcinoma [205], glioma [206], solid tumors [207], and hepatocellular carcinoma [208], among others.

We recently demonstrated the usefulness of using deep learning to analyze gene expression and classify images of celiac disease [77,78] and ulcerative colitis [173,184,209]. In this study, deep learning was used to classify LAIR1 image patches between celiac disease, small intestine control, and reactive tonsils. The proposed network managed to classify images with good performance. However, the aim was to conduct a proof-of-concept analysis, not to create a trained network production or commercial applications. Narrow artificial intelligence is not ready to take over the job of pathology-trained medical doctors because histological biopsies obtained from endoscopic examinations may be associated with other diseases. Notably, other research groups, such as Denholm et al., Molder et al., Scheppach et al., and Schreiber et al. (among others), have successfully used deep learning in celiac disease [210,211,212,213,214,215,216,217].

This study analyzed the phenotype of intraepithelial lymphocytes (IELs) and the lamina propria in the small intestine, including LAIR1, and confirmed LAIR1 expression in celiac disease. In celiac disease, both IELs and lamina propria cells were positive for LAIR1. Compared with the small intestine control, the lamina propria infiltration in celiac disease was higher. Finally, as a proof-of-concept AI analysis, a convolutional neural network classified LAIR1-stained image patches between the three diagnoses of small intestine control, celiac disease, and reactive tonsils with high accuracy. Therefore, IELs are positive for LAIR1. The LAIR1 marker is relevant in intestinal mucosa immunology and celiac disease.

Of note, one clinical trial targeting LAIR1 is listed on the website of clinicaltrials.gov (last accessed on 31 July 2025): A Safety, Tolerability and Efficacy Study of NC525 in Subjects With Advanced Myeloid Neoplasms (Id. NCT05787496, NextCure, Inc., drug NC525, Monoclonal antibody specific for LAIR-1) (NextCure, Inc., Beltsville, MD 20705, USA).

## 5. Conclusions

This study used several immuno-oncology and immune-phenotype markers to characterize the intraepithelial lymphocytes and the lamina propria of a small intestine control and to confirm the expression of LAIR1 in celiac disease. In small intestine mucosa control, IELs exhibited a cytotoxic T-cell phenotype and were CD3+, CD8+, CD103+, TCRβ+, and LAIR1+. In celiac disease, both IELs and many lamina propria cells were LAIR1-positive. In comparison to the small intestine control, LAIR1 lamina propria infiltration in celiac disease was higher. This study also successfully performed a proof-of-concept deep learning histological analysis of LAIR1 between the small intestine control, celiac disease, and reactive tonsils. A convolutional neural network classified LAIR1-stained image patches between the three diagnoses of small intestine control, celiac disease, and reactive tonsils with high accuracy (99.6%).

In conclusion, IELs are LAIR1+. High LAIR1 expression in IELs and lamina propria immune cells characterize CD.

## Figures and Tables

**Table 1:** Cells of villi and glandular crypts.

Cell Type	Function/Secretion
Intestinal epithelial cell	Nutrient absorption, infection protection, immune response
Paneth cell	Defensin secretion
Goblet cell	Adaptive immune response
Endocrine cells	
EC	Serotonin (5-HT) (gut motility)
L	GLP-1 (glucoregulatory), GLP-2, peptide YY (reduces appetite)
K	GIP (glucoregulatory, energy storage), 5-HT
I	Cholecystokinin (enzyme secretion, gallbladder contraction, and satiety), 5-HT
D	Somatostatin (inhibition of the production and secretion of other hormones, including glucagon and insulin)
G	Gastrin (stimulation gastric acid secretion)
N	Neurotensin (stimulation of intestinal mucosa growth)
M	Motilin (regulates gastrointestinal motility)
S	Secretin (regulation of pancreatic secretion, gastric acid release, and bile flow)

**Table 2:** Main properties of IELs.

CD103+
Express co-inhibitory molecules
Express NK receptors
Limited TCR diversity
Innate-like properties
Natural (n) IELs (thymus)
Peripheral (p) IELs (from CD4 or CD8+ cells)
Cytotoxic activity
(I) TCRγδ + nIELs (tissue surveillance and repair) (II) TCRαβ + CD8αα + nIELs (regulation) (III) TCRαβ + CD8αβ + pIELs (effector memory, cytotoxicity) (IV) TCRαβ + CD4 + pIELs (regulation, cytotoxicity)
CD8αα+ is an indication of intestinal IELs.

**Table 3:** Clinicopathological features of celiac disease.

Factors	Environmental	Gluten component of wheat and related cereals (gliadin) [99,100,101,102]
	Genetic	HLA-DR3-DQ2 and/or DR4-DQ8 [103,104,105,106,107,108] Non-HLA locus genes: *RGS1*, *IL18RAP*, *CCR3*, *IL12A*, *LPP*, *IL21*, *OLIG3*, *TNFAIP3*, *REL*, *TAGAP*, *SH2B3*, etc. (“immune-related genes/immunogenes”) [109,110,111,112,113,114,115,116,117,118,119]
	Mucosal immune response	Adaptive immune response (gliadin-reactive T-lymphocytes, autoantibodies) Intraepithelial lymphocytes (increased in active disease, with gammadelta T-cell receptor and with expression of interferon gamma and IL-10) Innate immune response (triggered by microbial components, chemicals, small molecules, and food-derived interleukin-15)
Epidemiology		Estimated 1% of global population based on serologic studies [120,121]
Phenotypes	Classic	Gluten-sensitive enteropathy that is characterized by diarrhea, malabsorption (including steatorrhea, weight loss, nutrient and vitamin deficiency), villous atrophy, antibodies against transglutaminase, and resolution of mucosal lesions and symptoms after dietary gliadin withdrawal [122,123].
	Nonclassic	Also known as atypical, with presence of extraintestinal manifestations but less malabsorption symptoms [124].
	Subclinical	Asymptomatic patients with only endoscopic or serologic findings.
	Potential	Patients with positive celiac-specific antibodies but normal mucosal biopsy; frequently found in children screened for celiac disease [125].
	Latent	Previously used term for patients who had celiac disease but recovered completely after a gluten-free diet [125].
	Refractory disease	Persistence of symptoms and villous atrophy despite gluten-free diet adherence [126,127,128,129]. This includes refractory celiac disease type 1 (RCD1), RCD2 (characterized by aberrant IELs with restricted gene rearrangements) [130,131,132], enteropathy-associated T-cell lymphoma (EATL) [81,82,83], collagenous sprue [133], and alternative diagnoses such as autoimmune enteropathy, common variable immunodeficiency (CVID), and drug-induced villous atrophy [134,135].
Clinical manifestations	Gastrointestinal	Usually diagnosed in children or young adults with classic signs of diarrhea and consequences of malabsorption, including weight loss, anemia, neurologic disorders (B-vitamin deficiency), and osteopenia (vitamin D and calcium deficiency).
	Extraintestinal	Mucocutaneous (dermatitis herpetiformis [136,137], atrophic glossitis) Metabolic bone disorders [138,139,140] Hematologic (iron deficiency [141,142], hyposplenism [143]) Elevated aminotransferases (mild to moderate chronic elevation of serum aminotransferases) Neuropsychiatric
Associated conditions	Selective IgA deficiency, autoimmune disease, gastrointestinal, menstrual, reproductive, idiopathic pulmonary hemosiderosis, cardiovascular disease, and kidney disease.	
Prognosis	Cancer risk	Increased risk of developing lymphoma [102] and gastrointestinal cancer [144].
	Mortality	Increased mortality [145].

**Table 4:** Intestinal lesions in celiac disease.

Subtype	Type 0	Type 1	Type 2	Type 3a	Type 3b	Type 3c
Histology	Pre-infiltrative	Infiltrative	Hyperplasic	Villous atrophy	Villous atrophy	Villous atrophy
Diagnostic lesions	No	No	Yes	Yes	Yes	Yes
Villi characteristics	Normal	Normal	Normal	Mild atrophy	Moderate atrophy	Severe atrophy
Crypt	Normal	Normal	Hyperplasia	Hyperplasia	Hyperplasia	Hyperplasia
Ratio, villus height–crypt depth	3:1	3:1	<3:1	<2:1	1:1	<1:1
IEL/100 EC	<40	>40	>40	>40	>40	>40

EC, epithelial cells (in the villi).

**Table 5:** Details of the primary antibodies.

Antibody	Company	Details
CD3	Leica	Mouse monoclonal, clone LN10, IgG1, C-terminal region
CD4	Leica	Mouse monoclonal, clone 4B12, IgG1, external domain
CD8	Leica	Mouse monoclonal, clone 4B11, IgG2b, alpha chain cytoplasmic portion
CD103 (ITGAE)	Leica	Rabbit monoclonal, clone EP206, IgG, residues of human CD103/ITGAE protein
Granzyme B (GZMB)	Leica	Mouse monoclonal, clone 11F1, IgG2a, N-terminus of the mature granzyme B molecule
TCR beta (β)	CST	Rabbit IgG, residues near the amino terminus of human TRBC1/TCRβ constant region 1 protein
TCR delta (δ)	CST	Rabbit IgG, total TRDC/TCRδ protein
CD56 (NCAM)	Leica	Mouse monoclonal, clone CD564, IgG2b, extracellular domain
CD16 (FCGR3A)	Leica	Mouse monoclonal, clone 2H7, IgG2a, external domain (both transmembrane and GPI-linked forms)
LAIR1 (CD305)	CNIO	Rat monoclonal, clone JAVI82A, IgG2a, k
PD-L1 (CD274)	Leica	Rabbit IgG, clone 73-10, C-terminal domain
PD1 (CD279)	CNIO	Mouse monoclonal, clone NAT105, IgG1
BTLA (CD272)	CNIO	Mouse monoclonal, clone FLO67B, IgG1
TOX2	CNIO	Rat monoclonal, clone TOM924D, IgG2a
HVEM (TNFRSF14)	Abcam	Rabbit polyclonal, IgG, exact immunogen is proprietary information
CD163	Leica	Mouse monoclonal, clone 10D6, IgG1, N-terminal region
HLA-DP-DQ-DR	CNIO	HLA-DP, DQ, and DR. Mouse monoclonal, clone JS76, IgG2a
IL4I1	CNIO	Interleukin 4 Induced 1. Rat monoclonal, clone BALI265E,543H,573B, IgG2a
FOXP3	CNIO	Mouse monoclonal, clone 236A, IgG1

CST, Cell Signaling Technology; Leica, Leica Biosystems K.K.; CNIO, Spanish National Cancer Research Center.

**Table 6:** Distribution of markers in control intestinal mucosa.

Antibody	Target/Pathway	IELs	LP
CD3	T-lymphocytes	High	High
CD4	Helper T-lymphocytes (including antigen-presenting cells)	Low	High
CD8	Cytotoxic T-lymphocytes	High	Low
CD103 (ITGAE)	Alpha E integrin & human mucosal lymphocyte antigen 1 (ITGAE), intraepithelial T-lymphocytes, FOXP3+ Tregs, CD4+ and CD8+ T cells, dendritic cells, and mast cells in mucosal tissues. Interacts with E-cadherin (epithelial cells)	High	High
Granzyme B (GZMB)	Lytic granules of cytotoxic T-lymphocytes (CTL) and in natural killer (NK) cells	Low	Low
TCR beta (β)	T-cell receptor	High	High
TCR delta (δ)	T-cell receptor	Low	Low
CD56 (NCAM)	Neurons, astrocytes, Schwann cells, NK cells, and a subset of activated T-lymphocytes	Low	High
CD16 (FCGR3A)	NK cells, granulocytes, activated macrophages, and subset of T cells (TCRαβ and TCRγδ)	Low	Low
LAIR1 (CD305)	Co-inhibitory receptor	High	High
PD-L1 (CD274)	Immune suppression and inhibition of T-cell activity	Low	High
PD1 (CD279)	Co-inhibitory receptor	Low	Moderate
BTLA (CD272)	Co-inhibitory receptor	Low	High
TOX2	Transcription factor, maturation of NK cells, and differentiation of T follicular helper (TFH) cells	Low	Moderate
HVEM (TNFRSF14)	Ligand of BTLA	Low	Low
CD163	M2-like macrophages	Low	High
HLA-DP-DQ-DR	Antigen presentation by APC	Low	High
IL4I1	APC, T-cell inhibition	Low	Moderate
FOXP3	Regulatory T-lymphocytes (Tregs)	Low	Moderate

IELs, intraepithelial lymphocytes; LP, lamina propria. High, moderate, and low must be interpreted as the expression of each marker within IELs and LP cells subsets in intestinal mucosa control.

**Table 7:** Correlation between the histological subtype and the Marsh classification.

	Marsh Histological Classification					
Type	0	2	3a	3b	3c	*p*-Value
Control	18/18 (100%)	0/18 (0%)	0/18 (0%)	0/18 (0%)	0/18 (0%)	<0.001
Celiac disease	0/16 (0%)	5/16 (31.3%)	6/16 (37.5%)	3/16 (18.8%)	2/16 (12.5%)	
Total	18/34 (52.9%)	5/34 (14.7%)	6/34 (17.6%)	3/34 (8.8%)	2/34 (5.9%)	

**Table 8:** Correlation between histological subtype and LAIR1 expression.

	LAIR1			
Type	Low (1+, <20%)	Intermediate (2+, 20–50%)	High (3+, >50%)	*p*-Value
Control	6/18 (33.3%)	12/18 (66.7%)	0/18 (0%)	0.004
Celiac disease	1/16 (6.3%)	8/16 (50%)	7/16 (43.8%)	
Total	7/34 (20.6%)	20/34 (58.8%)	7/34 (20.6%)	

**Table 9:** Correlation between Marsh classification and LAIR1 expression.

	LAIR1			
Marsh	Low (1+, <20%)	Intermediate (2+, 20–50%)	High (3+, >50%)	*p*-Value
0	6/18 (33.3%)	12/18 (66.7%)	0/18 (0%)	<0.001
2	1/5 (20%)	4/5 (80%)	0/5 (0%)	
3a	0/6 (0%)	4/6 (66.7%)	2/6 (33.3%)	
3b	0/3 (0%)	0/3 (0%)	3/3 (100%)	
3c	0/2 (0%)	0/2 (0%)	2/2 (100%)	
Total	7/34 (20.6%)	20/34 (58.8%)	7/34 (20.6%)	

## Data Availability

All data and methodology are available upon request to Dr. Joaquim Carreras (joaquim.carreras@tokai.ac.jp) and are also uploaded to the Zenodo open repository: Carreras, J. (2025). LAIR1 Celiac Disease for CNN (examples) (Version 1) [dataset]. Zenodo. https://doi.org/10.5281/zenodo.16451426; Carreras, J. (2025). LAIR1 images Celiac Disease and controls (Version 1) [dataset]. Zenodo. https://doi.org/10.5281/zenodo.16911678. Carreras, J. (2025). Cases of Celiac Disease (Version 1) [dataset]. Zenodo. https://doi.org/10.5281/zenodo.17014839.

## Supplementary Materials

The following supporting information (output file from CNN ResNet image classification) can be downloaded at: https://www.mdpi.com/article/10.3390/biomedicines13102526/s1.

## Abbreviations

The following abbreviations are used in this manuscript:

IELs	Intraepithelial lymphocytes
EATL	Enteropathy-associated T-cell lymphoma
LAIR1	Leukocyte-associated immunoglobulin-like receptor 1

## Appendix A

**Table A1:** Clinicopathological characteristics of patients with celiac disease.

Age	Sex	Biopsy Location	Diagnosis	Marsh	LAIR1
70	Male	Duodenum	Celiac Disease	3a	3+
62	Male	Pylorus/duodenum	Celiac Disease/Chronic gastritis	2	1+
62	Male	Duodenum	Celiac Disease	2	2+
78	Female	Duodenum	Celiac Disease	3b	3+
59	Male	Duodenum	Celiac Disease	3a	2+
44	Female	Duodenum	Celiac Disease	2	2+
17	Female	Duodenum	Celiac Disease	3b	3+
56	Female	Duodenum	Celiac Disease	3a	2+
54	Female	Duodenum	Celiac Disease	2	2+
58	Female	Duodenum	Celiac Disease	3b	3+
61	Female	Duodenum	Celiac Disease	3c	3+
45	Male	Duodenum	Celiac Disease	3a	2+
70	Female	Duodenum	Celiac Disease	2	2+
40	Female	Duodenum	Celiac Disease	3a	2+
61	Female	Duodenum	Celiac Disease	3c	3+
44	Female	Duodenum	Celiac Disease	3a	3+
63	Male	Small intestine control	Reactive lymphoid tissue	0	1+
64	Male	Small intestine control	Reactive lymphoid tissue	0	2+
64	Male	Small intestine control	Reactive lymphoid tissue	0	2+
64	Male	Small intestine control	Reactive lymphoid tissue	0	1+
72	Male	Small intestine control	Reactive lymphoid tissue	0	2+
72	Male	Small intestine control	Reactive lymphoid tissue	0	2+
63	Male	Small intestine control	Reactive lymphoid tissue	0	2+
63	Male	Small intestine control	Reactive lymphoid tissue	0	2+
68	Female	Small intestine control	Reactive lymphoid tissue	0	2+
68	Female	Small intestine control	Reactive lymphoid tissue	0	1+
63	Male	Small intestine control	Reactive lymphoid tissue	0	1+
53	Female	Small intestine control	Reactive lymphoid tissue	0	1+
64	Male	Small intestine control	Reactive lymphoid tissue	0	2+
73	Female	Small intestine control	Reactive lymphoid tissue	0	2+
73	Female	Small intestine control	Reactive lymphoid tissue	0	2+
73	Female	Small intestine control	Reactive lymphoid tissue	0	2+
76	Male	Small intestine control	Duodenum, reactive lymphoid tissue	0	2+
59	Male	Small intestine control	Jejunum, reactive lymphoid tissue	0	1+
55	Male	Tonsil (AI analysis)	Reactive lymphoid hyperplasia	N/A	N/A
51	Male	Lymph node (axilla)	Hodgkin lymphoma (IHC control *)	N/A	N/A
55	Male	Left testicle	Diffuse large B-cell lymphoma (IHC control *)	N/A	N/A
42	Male	Left testicle	Diffuse large B-cell lymphoma (IHC control *)	N/A	N/A
66	Female	Lymph node (neck)	Reactive lymphoid hyperplasia	N/A	N/A
28	Female	Tonsil	Reactive lymphoid hyperplasia	N/A	N/A
30	Female	Tonsil	Reactive lymphoid hyperplasia	N/A	N/A
28	Male	Tonsil	Reactive lymphoid hyperplasia	N/A	N/A
61	Female	Tonsil	Reactive lymphoid hyperplasia	N/A	N/A
45	Male	Tonsil	Reactive lymphoid hyperplasia	N/A	N/A
55	Female	Lymph node (neck)	Reactive lymphoid hyperplasia	N/A	N/A
26	Male	Tonsil	Reactive lymphoid hyperplasia	N/A	N/A
76	Female	Appendix	Reactive lymphoid hyperplasia	N/A	N/A
21	Male	Lymph node (abdomen)	Reactive lymphoid hyperplasia	N/A	N/A

Marsh, Marsh–Oberhuber classification; N/A, non-assessable/applicable; IHC, immunohistochemistry. * Not used for analysis, only as LAIR1 immunohistochemical staining internal control.
